# Corpus-based age of word acquisition: Does it support the validity of adult age-of-acquisition ratings?

**DOI:** 10.1371/journal.pone.0268504

**Published:** 2022-05-25

**Authors:** Filip Smolík, Maroš Filip

**Affiliations:** 1 Institute of Psychology of the Czech Academy of Sciences, Prague, Czech Republic; 2 Faculty of Arts, Charles University, Prague, Czech Republic; Centre National de la Recherche Scientifique, FRANCE

## Abstract

Age of acquisition (AoA) is presumed to reflect the age or relative order in which words are learned, but is often measured using adult ratings or adult-reported observations and might thus reflect more about the adult language than about the acquisition process. Objective AoA estimates are often limited to words whose referents can be shown in pictures. We created a corpus-derived AoA estimate based on first word occurrences in a longitudinal corpus of child English, and evaluated its reliability and validity against other measures of AoA. Then we used these different measures as concurrent predictors of adult lexical decision times. Our results showed adequate reliability and good relations with other AoA measures, especially with parent-reported AoA (r = 0.56). Corpus AoA did not predict unique variance in lexical decision times, while adult AoA ratings and parent-reported AoA did. We argue that this pattern is due to two factors. First, the adult AoA ratings and parent-reported AoA are confounded with adult memory, lexical processing and reading difficulty variables. Second, the adult AoA ratings are related to actual age of acquisition only for words acquired during later preschool and school age. Our analyses support the utility of corpus-derived AoA estimates as an objective measure of acquisition age, especially for early-acquired words.

## Introduction

Age of word acquisition is a variable that is related to the accuracy and time of processing in a number of linguistic tasks (see [1] for a review). In much of this research, the age of word acquisition is based on adult age-of-acquisition (AoA) ratings, in which adults provide their estimates of when they learned specific words or when these words are generally acquired by children. This indirect procedure correlates fairly well with other measures of age at which children learn words [2], but such validation measures have their limitations, too. Even though there is ample evidence of correlations between adult AoA ratings and various measures of early word knowledge, there is not much direct evidence that the adult ratings really reflect age when words are acquired and not some other word characteristics related both to acquisition age and processing measures [3, 4]. Additional direct measures of acquisition are thus useful. Such additional measures may help in understanding the nature of adult AoA estimates and their relation to the actual age of acquisition.

The present paper examines the estimates of word age of acquisition from longitudinal corpora of child language, and their relations to adult lexical decision times. While the corpus method is biased by frequency effects, this bias is likely to be different from biases in other existing AoA estimates. As a first step, we examine the relations between the corpus-based AoA and various other measures and estimates of AoA. Subsequently, we study how various AoA estimates relate to the lexical decision times in adults. Even though AoA accounts for a large proportion of variance in the lexical decision task [5], this relation could be inflated by confounds. This analysis examines whether the relations between adult AoA estimates and lexical decision times is likely to be due to the actual age of acquisition or rather to confounds stemming from adult language processing or representation.

### Age of acquisition effects

The age of word acquisition is related to various measures of word knowledge and processing in adulthood. In a review of the AoA research [1], the earliest paper found in this research area was by Rochford and Williams [6]. In this study, the age of word mastery, measured by successful picture naming, predicted the problems with word knowledge in patients with aphasia, with words acquired early preserved longer than words acquired later. Carroll and White [7] published the first study examining the effects of AoA in typical adults without impairments. One of the AoA measures used in this study were adult ratings of the age of acquisition, and the results showed that people were faster at naming pictures with words that had lower age of acquisition. A number of studies confirmed the effects of AoA on various measures of word processing [8–15]. An important suggestion was made by Morrison, Ellis, and Quinlan [16], who argued that the frequency effects in the picture naming task were in fact masked age-of-acquisition effects. Adults read the high-frequency words faster, but Morrisson et al. [16] suggested that this is not because they occur more often, but because they have been acquired earlier. Because AoA and frequency are correlated, the effects of frequency are found if AoA is not controlled for. In studies that used both word frequency and AoA as predictors of lexical decision times, some studies found no unique effects of frequency [12, 17], and others found independent effects of frequency and AoA [13, 18]. The relation between lexical decision times and the age (or order) of acquisition has been successfully modeled in connectionist networks [19–22]. Because the effect of frequency in word processing research is one of the most firmly established results in psycholinguistic research, the interplay between AoA and frequency in predicting response times is of considerable theoretical and methodological interest. An important assumption in this line of research is that the effects of AoA are interpreted as a relation with the actual age at which words are acquired, or the order in which words are learned in life. This interpretation crucially depends on the validity of the measures used to assess the AoA. However, the evidence of such validity is mostly indirect and is potentially confounded with a number of variables related to adult language representation and processing.

### AoA measures

The most common way of assessing the age of word acquisition is the use of adult AoA ratings. These are obtained by asking adults to provide estimates of when they learned each word from a list. Usually, a scale with some reference to chronological age is used; e.g. Carroll and White [7] used an 8-point scale where 1 meant 2 or 3 years, and 8 meant 14 years or more, with 1 point corresponding to two-year span. The instructions usually ask participants to rate when they learned the words in a list (for a review of the elicitation instructions, see [23, 24]). This means that AoA ratings are based on adult intuitions and perhaps informal observations of other children, not on the actual knowledge of the age when words are learned. People do not have good temporal recall of early childhood experiences [25, 26], which is likely to be the case for word acquisition as well. Bonin with colleagues [4] conducted experiments that challenged the notion that adults access chronological information of AoA when evaluating words. In their experiments, they found that typography influenced AoA ratings, suggesting that adults’ AoA ratings are affected by words’ surface features. They concluded that people do not have access to knowledge about word acquisition and rely on other information about words, such as frequency or familiarity. On the other hand Cortese and Khanna [27] claimed that when the interfering factors are controlled, it is possible to obtain unique effects of AoA on lexical processing. Controlling for many variables (including frequency), they demonstrated the AoA effect on lexical decision task and reading times. It is thus an open question to what extent AoA ratings reflect the actual age of acquiring words.

There are a number of studies that aimed to validate the AoA ratings. Most of them relied on the picture naming task in which children name pictures in a set of object and action pictures. The age at which the majority of children can name a picture can be interpreted as the age of acquisition estimate. The existing studies usually used one or both of two estimates: the age at which 75% children acquire the word, or the middle age of acquisition derived from logistic regression. Such estimates show moderate to strong correlations with the adult AoA estimates, with the correlation coefficients in the range of about 0.55 [28] to 0.75 [2]. The limitation of this method is that it always relies on a relatively small sample of words, typically 200 to 300. In addition, it must be possible to provide pictures of the words’ referents, which limits the range of words that can be tested. Most studies only used nouns labelling objects; to the author’s knowledge, only one set of picture naming data also includes action names [24]. Even with nouns, the method is only adequate for concrete nouns whose referents can be shown in pictures, while vocabulary contains many nouns that cannot be unequivocally pictured (e.g. brother, animal, play).

An alternative way of validating the AoA ratings is the use of parent-report data on children’s vocabulary. In instruments such as the MacArthur-Bates Communication Development Inventories (CDI) [29], adults are asked to check whether their child uses a word from a long list of words. The information reported by parents is generally viewed as reliable [29–31], and the parent-report vocabulary measures play an important role in current language acquisition research. The relation between adult AoA ratings and the parent-report data on word acquisition was examined by Łuniewska et al. [24] in 9 languages. The correlations between the AoA ratings and the percentage of children who used a word at a particular age ranged from 0.10 to -0.68 (mean -0.39). This suggests a moderate relation, but the values of correlation coefficients are difficult to compare with the previous studies, which based the objective AoA value on the age when 50% or 75% understand or produce a word. The use of parent reports has the advantage that it can be used even for relational, abstract or function words whose referents cannot be shown in pictures. The limitation of this method is that it only provides estimates for the set of words used in the parent-report instrument. This usually consists of several hundred words that are likely to appear in the language of children below 3. Such a sample is usually larger than in picture-based validations but by no means representative of the whole vocabulary, and biased towards early-learned words. An additional limitation of parent reports is that they depend on the parents’ ability to recognize words their children use, i.e. on recognition memory. Series of studies confirmed that rated AoA itself is related to memory [9, 32–34], and thus the two measures may be confounded with memory as a common third variable.

In addition to using picture naming and parent reports, other estimates of word mastery have also been used occasionally. Some studies asked children about the meaning of various words [35, 36]. Gilhooly and Gilhooly [37] used the order of item difficulty in a vocabulary scale based on verbal multiple-choice questions; Jorm [38] studied relations between AoA ratings in a single child and diary-records of word knowledge in this particular child. A special case is the set of test-based AoA norms published by Brysbaert and Biemiller [39]. These are based on data reported in a book by Dale and O’Rourke [40]. The norm set comprising more than 44000 word meanings was collected between 1950’s and 1970’s using multiple-choice questions in which each word was presented with three alternative meaning options. Children of different school grades marked their choices, and the word was assigned a grade level at which two thirds of children chose the correct response. Brysbaert and Biemiller [39] showed that the norms show good relations with adult AoA ratings, and that they are good predictors of adult lexical decision times. This is the most extensive and objective measure of age of acquisition but it should be noted that it is based on assessments of written word comprehension in children between grades 2 and 14, in two-grade steps (grade 2, 4, 6 and so forth). The granularity of this data set may be insufficient to distinguish between words acquired earlier in life, and its good relations with lexical decision data may be due to the fact that it is based on responses to written stimuli. It should also be noted that the data was collected 50 to 70 years age so it may not necessarily reflect current English.

A different approach to measuring AoA was brought by Zevin and Seidenberg with a frequency trajectory. This variable is based on the fact that some words are more frequent in childhood and not so much in adulthood, while others have the opposite characteristic or have a stable frequency throughout life. Zevin and Seidenberg [21, 41] proposed using this measure instead of the AoA measures and tested its validity in computational models and behavioural tasks. Some experiments show it has an effect on the estimation of AoA [21, 42] and it is a predictor for lexical processing [21, 41–43], but not in all tasks [21, 42, 44]. Frequency trajectory is not widely used in research, and Bayeen [45] argues that a better substitute for AoA ratings are ratings corrected for frequency. Brysbaert [46] performed a criterion validity analysis for both frequency trajectory and corrected AoA ratings, but found no evidence that they are a more accurate measure than classical AoA ratings.

To summarize, the majority of objective validations of AoA ratings is based on picture naming tasks, and most are limited to preselected samples of words with specific properties. The only extensive data set has some shortcomings of its own, particularly low granularity and the fact that it is based on written language, just like adult ratings. In order to examine the validity of adult AoA ratings more thoroughly, it would be useful to use other sources of objective data on word knowledge in children. This is the focus of the present paper.

### Longitudinal corpora and word emergence

In a world devoid of practical limitations, one could establish the age of acquisition by continuously observing the development of a group of children, noting the first usage of each new word, and recording the corresponding ages. However, this would require access to the majority of the children’s language productions, which is almost never the case (perhaps the own-family study by Deb Roy may be an exception [47]). The usual situation in research is that we have only occasional recordings, typically of a small group or single child. However, there are some longitudinal corpora that have regular and reasonably dense sampling over a longer period of time for a larger group of children. The present study uses the age of first-time occurrence in a longitudinal corpus as an objective estimate of the word’s age of acquisition, complementing the existing measures with a metrics that is most directly linked to early language acquisition in children.

To our knowledge, the corpus-based AoA has been previously used only by Grimm et al. [48, 49] (who label it as the age of first production—AoFP). Both studies investigated a similar topic, namely in what unit children’s language is segmented, with AoFP serving as a dependent variable tracking children’s word learning. In the first study [48], AoFP was regarded as the first usage of a word by any of the children in the American English corpora in the CHILDES database [50]. Strong positive correlation (Spearman’s rho = .61) was found between AoFP and AoA from a picture naming task [2], supporting the validity of this measure. Grimm et al. [49] used a slightly modified AoFP measure, also finding significant relations with picture-naming AoA (ρ = .65 or .59 for British and American corpora, respectively). These studies suggest that corpus based AoA is a useful estimate but Grimm et al. used it as a dependent variable and did not examine its validity in detail against different measures of AoA. The aim of our study is to examine closely these properties of the corpus based AoA.

The idea of using first-time occurrence is intuitive, but it is also burdened with a serious bias. Even if no learning occurs, the frequent words are more likely to be sampled earlier than words with lower frequency, which means that the age of first use is confounded with frequency. If a child uses a particular word very often, the word is likely to appear in the first recordings made with her. On the other hand, words used rarely may not occur at all, or appear late in the corpus because there was no opportunity or relevant context for their use previously, even though the word is known by the child from the beginning. However, even with this bias, the age of first occurrence is likely to capture some true variance in the actual age of acquisition. Words can only appear in children’s corpora if they have been acquired by children, so that the actual age of acquisition becomes the lower bound for observing a word in a corpus. Moreover, the frequency bias, however serious it may be, is likely to be different from the biases in other methods for establishing word age of acquisition. The majority of validation studies were based on picture naming and thus limited to concrete words, mostly nouns. The use of corpora has no such limitations, and will pick any words that children use with some minimum frequency. In addition, the corpus-based estimate is open, not based on a pre-selected set of words. It is thus not influenced by the expectations that drive the selection of words for the validation data, whether it is in a picture-naming study, in a study based on children questioning, or in a parent-report study. In contrast to parent-report studies, the corpus-based estimate does not depend on memory. Therefore, even though the frequency bias is a serious limitation for using corpus-based estimates, these estimates may provide important information for the interpretation of other estimation methods.

### Age of acquisition and adult lexical decision times

An important reason for studying the different AoA measures is that age of word acquisition is related to the adult performance on various word-related tasks. One of the most common tasks in adult psycholinguistics is the lexical decision task, measuring the time needed to classify a string as a meaningful word or a nonword. The primary variable affecting lexical decision times is word frequency. However, the AoA may also contribute to lexical decision times, with words that have lower AoA showing faster reaction times. A number of studies showed this effect [13, 51–56], although there are also opposite findings [12, 17]. The relations with lexical decision times suggest that age of acquisition is related to some aspect of word representation or processing in adults. There are three major proposals about why AoA might relate to adult lexical processing, all assuming that AoA reflects actual age at which a word is learned. One proposal is based on cumulative frequency: if a word is acquired early, an individual will be exposed to more occurrences of this word than of a word with comparable frequency that was acquired later [57]. The second proposal is based on semantics, suggesting that early-acquired words become prerequisites for the acquisition of later words, and are thus closer to the core of the semantic representational network [58]. The third proposal is based on neural network modelling, and the point is that neural networks lose their plasticity when incorporating more and more items [19]. However, given the indirect nature of many AoA estimates, it is possible that the relation to lexical decision times is due to factors that influence AoA but do not reflect the actual age of learning the words.

The most common AoA estimates, i.e. the adult AoA ratings or the parent reports, are mediated by adults and can be due to the properties of the adult language system. Adults’ responses in AoA ratings or in reports of word use by children may easily be influenced by factors that also affect lexical decision. For instance, lexical decision task is sensitive to the speed and efficiency of access to the words stored in long-term memory. At the same time, the adult AoA estimates may be influenced by the ability to recall own use of a certain word in childhood, or instances of observing children produce this word. Similarly, memory access may easily influence parental reports of word knowledge in children, as parental recall of the words their children say may be affected by the parent’s memory performance. In the case of the AoA estimates that use written materials, such as test-based AoA by [39], the confounding variables may also be various mechanisms related to reading. Because of these possible confounds in the relation between existing AoA measures and lexical decision task, it should be particularly informative to compare the concurrent relations of various AoA measures with lexical decision times, and this is analysis presented here.

### Goals and questions

The overall goal of this study was to examine whether the relations between different AoA measures and adult lexical decision times, examining whether different estimates of word age of acquisition provide valid and useful information on word acquisition. This goal includes examining the relations of AoA ratings with corpus-based age-of-acquisition estimates, and testing the relations between age-of-acquisition and lexical decision times. These goals were addressed in three steps. First, we calculated the corpus-based age-of-acquisition estimates from a longitudinal corpus of child language, including reliability estimates. Second, we examined the relations between these corpus-derived estimates and two traditional AoA measures, the adult ratings and the child data based on parental reports. Third, we used the lexical decision times to examine how different AoA estimates relate to word processing in adults, and whether such relations reflect any effects of early word acquisition.

The first step consisted of deriving the data from the Manchester corpus [59] available in CHILDES [60]. There are various ways in which the first occurrence of a word can be captured using a corpus that includes multiple children, and we thus created three different variants of the AoA estimate and examined their reliability.

The second step examined the relation between corpus-based AoA and the more traditional AoA measures, examining correlations between the corpus-based AoA estimates, estimates based on adult ratings, parent report and child testing. If corpus-based AoA relates to the other three measures to a similar extent that these measures relate to one another, the validity of the corpus-based AoA would be comparable to adult AoA ratings. If the corpus-based estimate has lower correlations with adult ratings and parent reports than these two AoA estimates have between them, it would suggest weaker validity. There are other possible scenarios, where corpus-based AoA is related to one of the criteria more strongly than to the other. These would have to be interpreted according to the exact pattern of results. It is also important to note that the interpretation is equivocal even when similar correlations are found between all three AoA measures. For instance, if there is a moderate relation between all three measures, it could mean that each of the three measures is related to the actual age of acquisition to the same extent. It could also mean that one measure is the most accurate and valid one, and that the two remaining measures each deviate from this best measure in different ways and suffer from different confounds. From the pattern of correlations only, it is impossible to decide which interpretation is appropriate.

In the third step, the different measures of AoA were examined as concurrent predictors of the adult lexical decision times in a multiple regression model, controlling also for word frequency. Because all measures are meant to estimate the age of word acquisition, considerable amount of overlap is expected between the effects of different predictors. If all predictors reflect the same underlying processes to a similar extent, it is possible that none of them will have a significant unique effect. On the other hand, if each AoA measure has a unique contribution, it would indicate that each is related to somewhat different aspects of lexical decision processes. If some predictors have significant unique effects while others do not, the significant predictors explain all the variance potentially explained by the rest, and their relation to lexical decision is thus arguably closer. Of special interest in these analyses was the corpus-based AoA estimate. A lack of unique effects of this estimate on lexical decision might suggest that the relations between lexical decision and other AoA estimates are due to confounds such as memory representation of words in adults, which is known to play a role in lexical decision [34, 61, 62]

## Method

### Corpus data on AoA

The corpus-based estimates of the word age of acquisition were based on the Manchester corpus. The corpus consists of 72 transcripts for each of the 12 children, made from recording on 36 different days approximately 10 to 14 days apart. Overall, the transcripts cover approximately the age range from 2 to 3, with some recordings before and after these points. In order to calculate the corpus-based AoA (corpus-AoA), a list of all different words used in the transcripts was generated for each transcript, using custom-written Perl routines. This search took into account only words spoken by the children. Then, the list was searched for the earliest occurrence of each word type in each child. This provided a list of words and their ages of first occurrence. Based on this, three different AoA values were calculated. One measure used the earliest occurrence in any of the children, regardless of how many children used the word. This means that even words that were only used by one child received an AoA value based on the age of first-time attested use in that child. This index is referred to as 1-child AoA. The second index is referred to as 6-child AoA, and includes words that were attested in six or more children. The AoA estimate is the mean age of the first-time usage from these six or more children. Finally, the 12-child AoA is the mean age of first occurrence for words that appeared in the transcripts of all 12 children.1-child AoA contains the largest number of words, but may contain a large number of idiosyncratic word occurrences, so it has limited reliability. However, 12-child AoA has these values reversed; it contains small number of words however it has better reliability. The 6-child AoA is somewhere in the middle. We performed our analyses using all three corpus AoA measures, so that we could compare whether they have common tendencies or differ in important ways. We made the resulting corpus available for use and stored it at OSF repository (https://osf.io/3dmpu/).

### AoA ratings, parent report data, and lexical decision times

The AoA ratings used in this paper were taken from the data set published by Kuperman, Stadthagen-Gonzalez and Brysbaert [63]. This large set contains norms for more than 30 thousand English words collected via Amazon Mechanical Turk. Rated words consist of nouns, adjectives and verbs taken from SUBTLEX-US corpus [64] and were not restricted in terms of frequency, or number of letters or phonemes. Each participant rated 300 words by writing the age in years when they thought they first learned the word, in the meaning that they understood the word even if they did not actively use its. The authors report that the set is fully comparable with previously reported sets of AoA norms that were collected under laboratory conditions. The respondents resided in U.S., while our other variables were based on British data, but we decided to use the US data set because it is much larger than any specifically British norms. Word frequency data was obtain from the SUBTLEX-UK database, which includes also children frequencies [65, 66].

Parent report data on children’s use of words were obtained from the British English standardization data for MacArthur-Bates CDI [67] that is available on Wordbank [68]. The age of acquisition was calculated for each word using a general linear binomial model with the logit link. The model estimated the relation between age and the proportion of children who are reported to say the given word. Based on the estimates, the expected age at which half of the children say the word was calculated (i. e. when the logit of this proportion is equal to zero). This was used as the objective age of acquisition estimate.

The test-based AoA were taken from supplementary materials to Brysbaert and Biemiller [39]. Each word in this set was assigned a value between 2 and 14 in steps of 2, except that the number 13 was also included. These numbers corresponded to the grade levels. The norm set contains different meanings for a number of words, with potentially different AoA values. For these words, the different meanings were merged and the lowest AoA value was used.

Lexical decision times were obtained from the British Lexicon Project [69], a data set of lexical decision times on more than 28000 English words collected in Britain. This data set was collected from two groups of 40 participants, each of whom responded to a half of the total number of words, about 14000. The British database of lexical decision times was chosen because it reflects the variety of English used in the Manchester corpus, the main source of child data here.

## Results

The extraction of corpus-based AoA first identified 4689 different words that appeared at least once across the 12 children. Many of these words appeared in multiple children, but some in one child only. Counting the first occurrence in each child separately, this resulted in 16266 unique first word occurrences. When only words that occurred six or more children were included, there were a total of 1054 different words; since each word had to be acquired by at least 6 children, there were 10135 unique first occurrences in an individual. Counting only words that appeared in all 12 children, there were 360 words with 4320 first occurrences. Based on these data, the 1-child, 6-child, and 12-child age of acquisition indices were calculated for the words that occurred by taking the mean age of first occurrence in the children who used the word.

### Reliability of corpus AoA

Intraclass correlation (ICC) and split-half method were used to measure the reliability of this corpus-based AoA measurement. ICC was measured as inter-rater reliability, with children serving as raters, and words as rated subjects. Two-way random effects model for the average of all ratings was chosen (ICC2k), where both raters and rated subjects are viewed as random effects, and where the mean value of all raters is taken as the assessment basis for the following analysis. ICC for 12-child and 6-child corpus AoA found good reliability, 0.76 and 0.83, respectively. For the split-half reliability, children were divided into two halves, and the correlation of mean age of acquisition was measured between the two groups. This method also shows good values for both measurements, 0.79 for 12-child AoA and 0.67 for 6-child AoA. The reliability of 1-child corpus AoA can only be measured in the form of split-half reliability, not in the form of internal consistency. However, in order to measure reliability even with split-half method, we need at least 2 values for each word, which was not possible to calculate for words spoken by only one child. This means, split-half reliability for 1-child AoA was measured for words where the word was produced by two or more children. As expected, the 1-child AoA reliability had the lowest value of 0.49, which lies on the borderline of good reliability.

### Relations between different AoA estimates

In the first analysis, the relations between corpus-based AoA, the adult AoA ratings and the test-based AoA were examined. This initial analysis did not include the AoA estimates based on parent report, so that the data set is not reduced to this relatively small group of words, and all available observations on corpus-based AoA are used.

**Fig 1:**
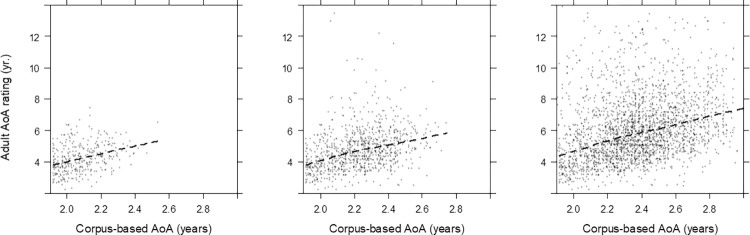
Relations between corpus-based AoA estimates and the adult AoA estimates. From left to right on x-axis is 12-child, 6-child, and 1-child word set.

Fig 1 shows the relation between these two variables without any adjustments separately for the 12-child, 6-child, and 1-child corpus AoA values. There is a linear relation between the measures with a similar slope in each corpus. At the same time, it is clear that the adult ratings estimate the age of acquisition as much higher than the first word occurrences in child corpus suggest.

Because the corpus-based AoA is likely to be severely biased by word frequency, further examination also included log-frequency of the words, and the frequency-adjusted corpus AoA, which was the residual after regressing corpus-based AoA on child log-frequency in the whole sample of words from the child corpora. Child frequency was calculated as the mean of the frequencies from preschool (0–6 years) and primary school children (6–12 years) from the British SUBTLEX database [65].

The results are summarized in Table 1. The correlation between corpus AoA and adult-rated AoA was between 0.30 and 0.32 in the three sets of words. This is remarkably consistent moderate correlation. The correlation between frequency and corpus-based AoA was also consistent across word sets, ranging from -0.30 to -0.36. However, the relation between corpus-based AoA adjusted for frequency and the adult AoA ratings differed across word sets. In the smallest, 12-child word set, the correlation was 0.32, i.e. similar to that with the unadjusted corpus-based AoA. In the 6-child and 1-child word sets, the correlation was 0.21 and 0.12, respectively, which suggests that the larger word sets were subject to increasingly stronger frequency bias.

**Table 1:** Correlations between adult AoA ratings, raw and adjusted corpus AoA estimates, and children frequency.

	AoA rating	Corpus AoA	Adj. corpus AoA	Test AoA
	12-child word set (334 words)			
Corpus AoA	0.31			
Adj. corpus AoA	0.32	0.83		
Test AoA	0.18	0.06	0.03	
Child log-frequency	0.01	-0.30	0.28	-0.04
	6-child word set (892 words)			
Corpus AoA	0.32			
Adj. corpus AoA	0.21	0.91		
Test AoA	0.55	0.11	0.02	
Child log-frequency	-0.29	-0.36	0.06	-0.22
	1-child word set (2344 words)			
Corpus AoA	0.30			
Adj. corpus AoA	0.12	0.94		
Test AoA	0.65	0.15	0.01	
Child Log-frequency	-0.54	-0.34	-0.00	-0.40

The relations between test-based AoA and corpus AoA were practically zero, especially for the frequency-adjusted corpus AoA. On the other hand, the test-based AoA and adult AoA ratings showed a strong correlation (r = 0.65) in the 1-child word set, although the relation was much weaker in the smaller 12-child set.

**Fig 2:**
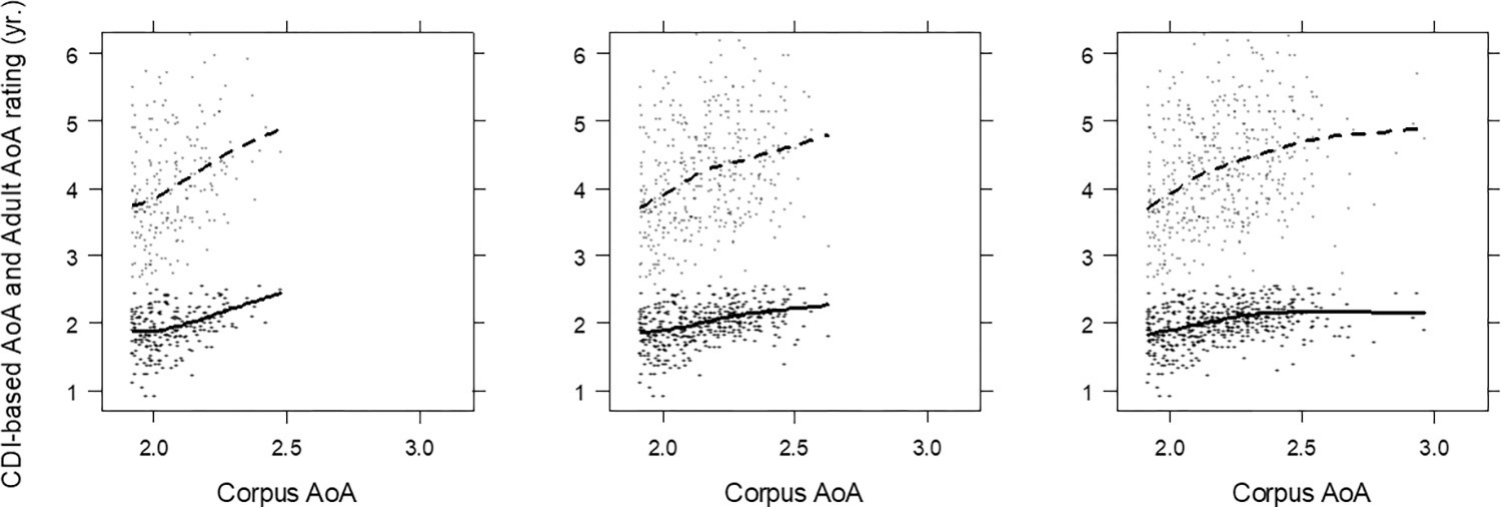
Relations between corpus-based AoA, adult AoA, and parent-report AoA estimates. From left to right on x-axis is depicted corpus based AoA 12-child, 6-child, and 1-child word set, respectively; on y-axis in dashed line is adult AoA estimates, and in solid line parent-report AoA estimates.

The relations between corpus-based and parent-report AoA were examined in the next step, which included the AoA estimates based on the MacArthur-Bates CDI. The set of words in these estimates is smaller than for the estimates reported in Table 1, because the British English CDI data on Wordbank include 645 words, and there was only partial overlap between the Wordbank data and the corpus-based data sets. The results are summarized in Fig 2 and Table 2. The figure shows both parent-report AoA and adult rating AoA as a function of corpus AoA. The adult ratings are included to show their relation to frequency-adjusted corpus AoA within the smaller set of words overlapping with parent-report data.

**Table 2:** Correlations between parent-report AoA, raw and adjusted corpus AoA, and adult AoA ratings.

	AoA rating	Corpus AoA	Adj. corpus AoA	Parent report	Test AoA
	12-child word set (216 words)				
Corpus AoA	0.31				
Adj. corpus AoA	0.37	0.82			
Parent report	0.56	0.38	0.61		
Test AoA	0.19	-0.03	-0.01	0.13	
Child log-frequency	0.07	-0.34	0.25	0.37	0.05
	6-child word set (386 words)				
Corpus AoA	0.31				
Adj. corpus AoA	0.33	0.90			
Parent report	0.56	0.42	0.56		
Test AoA	0.14	0.05	0.06	0.14	
Child log-frequency	-0.04	-0.45	-0.01	0.17	0.01
	1-child word set (456 words)				
Corpus AoA	0.31				
Adj. corpus AoA	0.28	0.92			
Parent report	0.55	0.36	0.44		
Test AoA	0.18	0.07	0.05	0.08	
Child log-frequency	-0.15	-0.50	-0.12	0.07	-0.06

All within the subset of words present in the parent-report instrument.

Table 2 shows that the correlation between corpus-based AoA and the AoA based on CDI is similar across the three data sets, between 0.36 and 0.42. When corpus-based AoA is adjusted for frequency, the pattern is different, and the correlation becomes stronger in the smaller sets. With all first occurrences from the corpus used, the correlation with CDI-based AoA is 0.44, but it is 0.56 with the 6-child data set, and 0.61 with the 12-child data. In other words, when the analysis becomes more and more focused on the words that are universally present in young children, the relation between corpus-based AoA and AoA based on parent reports (CDI) becomes stronger. With the 12-child data set, the correlation between corpus-based AoA and the CDI-based AoA is almost as strong as that between parent-reported and adult-rated AoA (0.61 and 0.56, respectively).

The test-based AoA again showed no meaningful correlations with corpus AoA, raw or adjusted, with r’s ranging from -0.03 to 0.07. Also the correlations between parent report and test-based AoA were weak, between 0.08 and 0.14. This suggests that test-based AoA is not a good indicator of acquisition age for words acquired early in childhood.

In addition to correlations, regression analyses were used to examine the concurrent effects of adult AoA ratings and corpus-based AoA estimates on the parent-report AoA estimates. The parent-reported AoA was used as the criterion because it may be viewed as unquestionably related to children’s word acquisition, and therefore can serve to validate the other two measures and their individual contributions. The results suggest that each has clear unique contributions. Adult AoA ratings and frequency-adjusted corpus AoA accounted for a similar amount of unique variance in parent-report (CDI) estimates using the 12-child word set (16.4% for corpus-based AoA, and 12.9% for AoA ratings, both p’s<0.001), as well as in the 6-child set (13.5% corpus-based AoA, 16.0% for AoA ratings, both p’s<0.001). In the 1-child word set, there was a clear difference with stronger effects for the AoA ratings (8.1% for corpus-based AoA, and 18.6% for the AoA ratings, both p’s<0.001), but each of the factors still had an independent effect. The test-based AoA values had no significant effect above and beyond adult and corpus AoA estimates in any of the word sets (p values 0.20, 0.10 and 0.64 for the 12-child, 6-child and 1-child sets, respectively). Variance inflation factor was used to control for multicollinearity, with no sign of undue effect (VIF ranged from 1.04 to 2.38). These results showed that the corpus-based AoA contains information uniquely related to the parent-reported objective age of acquisition, and may thus provide additional information over and above the adult AoA ratings. The test-based AoA, on the other hand, does not seem to be specifically related to parent-reported AoA, suggesting that this measure does not tap in the age of acquisition for early acquired words.

### AoA estimates and adult lexical decision times

The comparison of the four ways of addressing AoA (adult ratings, parent report, testing and child corpus) suggested that the measures share mutual relations that overlap to some extent but are partially unique to each pair of variables. This leads to the question of which of these measures is most relevant for predicting the processing of words in adults. This was addressed by examining the effects of the various age-of-acquisition variables on adult lexical decision times.

Two analyses were performed with lexical decision times as the dependent variable. In the first analysis, three AoA measures were used as predictors, the corpus-based AoA, test-based AoA and adult AoA ratings. Following Brysbaert’s recommendations [46], we used the corpus AoA estimate not corrected for frequency, but we also checked these analyses using the frequency-corrected AoA. The parent-report AoA was excluded to keep the sample of words as large as possible. In addition, the models controlled for adult word log-frequency. In the second analysis, all four AoA estimates were used as predictors, along with frequency. All analyses were performed separately for the three corpus AoA criteria.

The standardized regression coefficients from the first model are summarized in Table 3. In all three word sets, the effect of adult AoA ratings was statistically significant with larger effect sizes increasing with decreasing sample size (β = 0.33 in the 12-child set and β = 0.19 in the 1-child set). The corpus-based AoA, on the other hand, had no significant effect in any of the models, with estimated values around zero. This was the case also when frequency-corrected AoA was used. The effect of test AoA was only significant in the 6- and 1-child sets (β = 0.13 and 0.16), although the estimated magnitude was largest in the 12-child data set (β = 0.17). The only significant difference between model with corrected and uncorrected corpus AoA was the significant effect of frequency in 12-child set for corrected model (β = -0.08, p = 0.042) in comparison to the uncorrected model (β = -0.06, p = 0.083). But the effect got stronger with the size of the word set, with β of -0.20, and -0.45 for the 6-child, and 1-child word sets, respectively. This is not surprising because the larger word sets provide more variability against which the relation between frequency and response times can show. To examine the possibility of overfitting, we performed a 5-fold cross-validation with 4 repeats, with RMSE between 0.61 and 0.76, while the dependent variable values ranged from 471 to 690. No indication of overfitting is thus present.

**Table 3:** Standardized coefficients from regression analyses examining the effects of AoA predictors on lexical decision times.

	12-child set		6-child set		1-child set	
Predictors	β	p	β	p	β	p
(Intercept)	-0.10	0.432	-0.13	**<0.001**	-0.00	1.000
Corpus AoA	0.04	0.601	-0.05	0.120	-0.03	0.080
Adult rating AoA	0.33	**<0.001**	0.26	**<0.001**	0.19	**<0.001**
Test-based AoA	0.17	0.326	0.13	**0.009**	0.16	**<0.001**
Log-frequency	-0.06	0.083	-0.20	**<0.001**	-0.45	**<0.001**
Observations	325		839		2115	
R^2^ / R^2^ adjusted	0.080 / 0.069		0.200 / 0.196		0.426 / 0.425	

In the second step, the analyses were repeated with the AoA based on parent reports (MacArthur-Bates CDI) as an additional predictor (Table 4). This limited the analyses to a substantially smaller set of words. Frequency, adult AoA and parent-report ratings had significant effects on response times in all three analyses. For frequency, the effect got stronger in the larger data sets (β = -0.17 in the 12-child set and -0.39 in the 1-child set). The effect showed no clear tendency for adult AoA (β varies from .14 to .23) and parent report AoA (β varies from 0.13 to 0.21). Corpus-based AoA had a significant effect in the 6-child word set, but in the unexpected direction. The test-based AoA had no significant effect in any of the word sets. When we used the frequency-corrected corpus AoA, there were anymore no significant effect of Parent report AoA (p = 0.053) and log frequency (p = 0.074) in 12-child set. Overall, there is no evidence that corpus- or test-based AoA are related to adult lexical decision times, after taking into account adult-estimated AoA and word frequency. On the other hand, parent report AoA shows reliable, even though weak, relations to lexical decision times. Cross-validation provided RMSE values from 0.82 to 0.91, with dependent variable ranging from 471 to 690, thus showing no signs of overfitting.

**Table 4:** Standardized regression estimates for models predicting lexical decision times using Corpus AoA, adult AoA ratings, and log-frequency.

	12-child set		6-child set		1-child set	
Predictors	β	p	β	p	β	p
(Intercept)	-0.11	0.207	-0.06	0.165	-0.00	1.000
Corpus AoA	-0.13	0.258	-0.17	**0.012**	-0.04	0.143
Adult rating AoA	0.23	**0.002**	0.14	**0.011**	0.15	**0.003**
Test-based AoA	0.23	0.167	0.01	0.911	-0.02	0.697
Parent report AoA	0.17	**0.034**	0.21	**0.001**	0.13	**0.022**
Log-frequency	-0.17	**0.032**	-0.33	**<0.001**	-0.39	**<0.001**
Observations	216		386		456	
R^2^ / R^2^ adjusted	0.145/ 0.124		0.131 / 0.120		0.177 / 0.168	

## Discussion

The main goal of the present study was to compare different measures for the age of word acquisition and evaluate them against adult lexical decision times. A particular focus was on a measure of acquisition age derived from a child language corpus. The results suggest that there is considerable overlap between most of the measures of acquisition age. The corpus-based measure of acquisition age shows good relations with adult estimates and especially with parent-report data, but it has no relation to the test-based estimates and no unique relation to the adult lexical decision times. This could mean that the corpus-based measure is imprecise to the extent that it is not valid for studying the adult vocabulary. However, the pattern of findings is consistent with the view that the relations between existing measures of AoA and adult lexical decision times are due to confounding factors other than the actual age of word acquisition. We argue that latter possibility is correct, and that the findings presented here indicate weaknesses of the adult age-of-acquisition estimates. This appears to be particularly true for words that are acquired early in life.

### The corpus-based AoA estimate

The first step in addressing the goals of this study was to examine relations between four measures of word age of acquisition. This included deriving a measure of acquisition age that has not been widely used before, and examining its validity and reliability. A striking finding is how high the adult-estimated ages of acquisition are for many words that were found in the children’s corpus. This is the case even for words that were used by at least 6 or all 12 children in the sample, which cannot be viewed as outliers, such as family-specific words or words used in rhymes and songs. In the 12-child set, there were 75 words out of 334 with rated age of acquisition 5 or above (including words *away*, *back*, *hole* or *shop*), and in the 6-child set, a total of 134 words (of 897) had AoA ratings 6 or above. The actual upper age included in these data sets was about 2 years and 8 months. The difference is even more striking because the adult norms asked about the age of first comprehension, while the corpus-based data are on production, which normally lags behind comprehension.

The discrepancy might be viewed as an issue of scaling. Bonin and colleagues [4] noted that people are likely to use some subjective experience when approaching the rating task. They draw on the concept of “fluency” [70], which refers to the ease of completing a mental task. Thus, people might rate words based on their fluency, i.e., shorter, more frequent and more imageable words are rated as mastered earlier than their opposites. Perhaps adults, on average, misjudge the exact age at which words are learned but have good estimate of the relative difficulty of words. However, such misjudgement alone would be sufficient to question the accuracy of adult AoA ratings. A systematic shift in the estimated acquisition age towards higher ages suggests that the ratings are not based on people’s early memory or observations of children. The correlations with other measures can be explained by confounding factors, such as people’s beliefs about language acquisition, accessibility of words in adult memory or factors related to reading comprehension.

The relations between different AoA estimates in our data provide other suggestions. The frequency-adjusted corpus-based AoA shows stronger relation to the estimates based on parent reports (up to r = 0.61) than adult AoA ratings (highest r = 0.37). The parent-reported AoA values are likely to reflect the actual acquisition age better than the adult ratings, and because the corpus-based estimate is more strongly related to parent report than to the adult estimates, it appears that the adult AoA estimates are less precise measures of the real age of acquisition, compared to corpus-based and parent-reported data.

An intriguing set of findings was produced by examining the test-based AoA from school-age children. There were no relations between corpus AoA and test-based AoA, potentially undermining the validity of corpus AoA. But the relations between corpus AoA and parent-reported AoA were solid, while the relations between parent-reported and test-based AoA were weak or absent (highest r = .14). Test-based estimates showed good relations with adult AoA ratings, especially in larger word sets such as the 1-child set without parent report data. This pattern suggests that test-based AoA is more similar to adult ratings than to the parent-reported and corpus AoA that focus on words acquired early in life. This is not surprising given that the lowest age of acquisition in the test-based norms by Brysbaert and Biemiller [39] is grade 2, i. e. around 8 years. By that age, the words that occur in 2-year-olds should be already acquired, and this should result in low or zero correlation. The data are in line with this: of the 325 words in which the 12-child corpus word set and the test-based AoA set overlapped, only 18 were reported as acquired at grade 4, while the remaining 307 at grade 2.

On the other hand, it should be noted that for the words that occurred in the longitudinal corpus for 6 or more children, there were 123 of 839 words with test-based AoA at grade 4, i. e. 10 years, or higher. This shows that a number of words that are fairly common in 2-year-olds have test-based AoA at 10 years of above. So, the test AoA shows similar upward shift in estimated age as the adult AoA estimates. Perhaps responding to the test-based AoA does not only include the knowledge of the word and ability to use it, but also the ability to comprehend the written description of its meaning. In any case, the accuracy of test AoA for words acquired early in life is questionable.

The test-based AoA data and the adult AoA ratings are available for a much wider range of words than the estimates based on parent-report and the corpus-based estimates. The comparison of the four types of AoA estimates is limited to the words that are available for all of them, which in this case means words that appear in children before the age of about 3. The moderate-to-low correlations with corpus-based AoA might stem just from the limited variability in the sample of words, resulting in lower correlations. This is certainly an important factor; the adult AoA ratings are often used with words that are learned later in the school age, and some studies using AoA explicitly interpret the AoA measure as the age at which children are exposed to words in print [71]. However, the current results show that many words that are commonly considered as school age acquisitions are in fact routinely used before the age of 3. If the AoA ratings show this level of imprecision for words acquired early in life, there is not much reason to suppose the ratings will be more precise for words acquired later. The adult AoA ratings and test-based AoA should thus be used very carefully for arguing about the lasting effects of learning age or learning order. In particular, these measures must be interpreted as indicators of differences between words acquired at different stages during school age, rather than differences between acquisition timing in the early stages of building a vocabulary. This is important for the theoretical implications of any observed AoA effects; these should not be interpreted as consequences of events during the earliest stages of lexical acquisition. And it is quite possible that the relations between the actual age of acquisition and its adult rating are due to factors other than the acquisition age, such as the accessibility in memory, fluency of the words or reading difficulty.

### AoA and lexical decision times

Second important finding in the present study is that while the adult AoA ratings and parent-report AoA estimates have unique independent relations with adult lexical decision times, the corpus-based AoA are absent or possibly have the unexpected direction. The test-based AoA estimates only have significant relations with lexical decision times in the larger data sets, the 6-child and 1-child sets without parent report data. This is consistent with the idea that test-based AoA reflects differences between words acquired across the span of school age but it does not differentiate between words acquired earlier. At the same time, the significant relation between parent-reported AoA and lexical decision times shows that the restricted size of the word set does not preclude finding significant relations.

The absence of corpus-based AoA effects on lexical decision could mean that the measure is not related to the actual age of acquisition. However, the correlations between different AoA measures support the validity of the corpus-based estimates, especially against the parent-reported AoA, which is a more direct measure of acquisition age than the adult ratings. It would be difficult to explain these correlations if corpus-based AoA was too noisy to reflect the actual age of acquisition.

It is more likely that corpus-based AoA is a valid measure of word acquisition age but that the relations between other AoA measures and the lexical decision times are due to other factors that also influence adult ratings and parent reports, such as memory, which may affect adult AoA judgments, parent reports, as well the lexical decision times. Test-based AoA, besides its low granularity at lower ages, is mediated by reading processes, which obviously participate in lexical decision times. Other potential confounds that may be responsible here include familiarity, phonological complexity, emotional valency, objective or subjective frequency, and many others. Most of these factors can be included under the previously mentioned term of fluency. If people generally rate disfluent words to be later acquired, there will be a natural relationship between adult AoA and the lexical decision task. Since less fluent (longer, less frequent, more abstract, etc.) words are actually acquired mostly later, this may explain the correlation between adult AoA and other AoA methods. This relationship should represent order of learned words rather than the exact age, which is consistent with our findings.

However, if adult AoA ratings are related to parent report and lexical decision times due to confounding variables, why do they show validity against objective measures of AoA based on picture-naming [2, 28] and verbal explanation tasks [35, 37]? One possibility is that these validation studies are typically limited to nouns and have to include content that can be shown in pictures. Adult estimates of acquisition age may be more accurate for this type of words compared to non-picturable words such as relational terms. It is also possible that the age of acquisition does in fact relate to lexical decision times, but as discussed above, on a different scale than the range of words analyzed here. Even if the differences captured by the corpus-based AoA measure are real, they may be too small to have effects on lexical decision times. Perhaps the age of acquisition only makes large enough difference in response times if the difference in age is several years, not several months, as it is here. This, again, argues against any interpretation of the age-of-acquisition effects rooted in the earliest stages of lexical acquisition.

Unlike our study, Grimm et al. [48] found a significant correlation between their version of corpus AoA estimate and lexical decision times (Spearman’s ρ = 0.31). However, their correlation was based on 10,883 words, compared to 2117 in our largest corpus. Because Grimm et al. used a larger and more diverse set of transcripts to derive their AoA, their corpus AoA values cover a wider age span. Their finding is thus consistent with the view that age of acquisition is related to lexical decision times for words acquired later in childhood, not in the earliest stages. Another important note is that we tested the prediction value of the corpus AoA only in the confines of the lexical decision task. AoA plays a role in a variety of linguistic processes and to a different degrees; future analyses involving different tasks (e.g., written) should follow to determine the overall and more precise impact on language processing.

The pattern of results shown in this paper suggests the age of acquisition may be a deceptively complex variable. Researchers who use it in their work should take care to clarify their understanding of the concept. One aspect is the age at which a word becomes understandable to the child, and the child can reliably distinguish its meaning from other concepts. The other is the age at which a child can use the word adequately in her or his own productions, perhaps not necessarily with full understanding of the meaning but with solid enough representation to avoid misunderstandings or obvious errors. These two conceptions of age of acquisition can be viewed as complementary, but they reflect different aspects of word learning process, and the theoretical explanations of age-of-acquisition effects in language processing do not seem to make this distinction. The corpus-based age-of-acquisition data presented in this paper suggest that many words appear in children much earlier than their AoA ratings or even child testing suggests. We should thus use caution when interpreting the relations between ratings and test results and adult processing as consequences of the acquisition age or order.

## Summary and conclusions

Different measures of word age of acquisition show sufficient amount of overlap to assume that they reflect similar phenomena, but there is an important distinction between measures focused on words acquired early in life and those acquired in preschool and school age. The corpus-based age-of-acquisition measure presented in this paper can provide meaningful and valid data about the acquisition of words early in life, and suggests that these data can complement the existing ways of estimating AoA. At the same time, we show that many words estimated by adults as acquired during school age in fact appear before the age of 2.8. The interpretation of adult AoA ratings and test-based estimates should thus be very careful. It may only be appropriate for estimating the age of acquisition of words acquired later in childhood, and the relations between adult AoA ratings and measures of word acquisition may be to a large extent due to confounds.

Corpus-based AoAs have stronger relations with parents’ reports of word acquisition, which is the AoA measure closest to the actual age of adoption. The estimates based on words occurring in all 12 children show correlation with parent-report based AoA (r = 0.58) that is similar to correlations between adult AoA ratings and the picture-naming tasks. The corpus-based AoA estimates should thus be viewed as a useful measure that is subject to different types of biases than other estimates, with special value for examining words acquired in early stages of language development.

There was no relation between adult lexical decision times and the corpus-based AoA estimates. While this could question the validity of corpus AoA estimates, the pattern of findings here indicates that previously observed age-of-acquisition effects in lexical decision might be due to confounding factors affecting the AoA measures previously used. These are mediated by adult judgments and reading processes which can become confounding factors. This is another indication in our study that the widely used adult AoA ratings may be strongly influenced by the adult language system and have little validity for estimating age of word acquisition, particularly for words acquired in early childhood.
